# Supplementary material for: Thermal Insulation Foam of Polystyrene/Expanded Graphite Composite with Reduced Radiation and Conduction
Source: Polymers (Basel). 2025 Apr 11;17(8):1040. doi: 10.3390/polym17081040 (PMC12030753; doi:10.3390/polym17081040)
Supplement: Supplementary file 1 [file polymers-17-01040-s001.zip › polymers-3408489-supplementary.pdf]

SUPPORTING INFORMATION

Thermal Insulation Foam of Polystyrene/Expanded Graphite Composite with Reduced Radiation and Conduction

Pengjian Gong[1,2], Minh-Phuong Tran[1], Piyapong Buahom[1], Christophe Detrembleur[3,4], Jean-Michel Thomassin[3], Samuel Kenig[5], Quanbing Wang[6], Chul B. Park[a,*]

[1] Microcellular Plastics Manufacturing Laboratory (MPML), Department of Mechanical and Industrial Engineering, University of Toronto, 5 King's College Road, Toronto, ON M5S 3G8, Canada; pgong@scu.edu.cn (P.G.); minh.phuong.tran.fr@gmail.com (M.-P.T.); piyapong@mie.utoronto.ca (P.B.)

[2] College of Polymer Science and Engineering, Sichuan University, 24 Yihuan Road, Nanyiduan, Chengdu 610065, China

[3] Center of Education and Research on Macromolecules (CERM), CESAM Research Unit, Department of Chemistry, University of Liege, Allée de la Chimie, B6a, Sart Tilman, 4000 Liège, Belgium; christophe.detrembleur@ulg.ac.be (C.D.); jean-michel.thomassin@celabor.be (J.-M.T.)

[4] WEL Research Institute, 1300 Wavre, Belgium

[5] Department of Polymers and Plastics Engineering, Shenkar College, Anne Frank Street 12, Ramat Gan 52526, Israel; samkenig@shenkar.ac.il

[6] Jiangxi Tongyi Polymer Material Technology Co., Ltd., Jiangxi Tongyi New Material Industrial Park, Xinfeng County, Ganzhou City, 341600, China; wangquanbing@tongyiplastic.com

* Correspondence: park@mie.utoronto.ca; Tel.: +1-416-978-3053

Expanded Graphite Morphology

The morphology of EG characterized by scanning electronic microscopy (SEM) and transmission electronic microscopy (TEM) was shown in Figure S1. And the morphology of EG dispersed in PS/EG foam characterized by optical microscopy was shown in Figure S2.

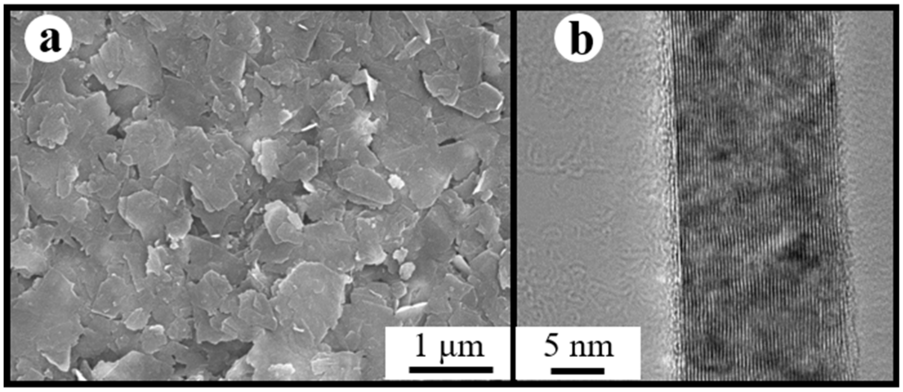

Figure S1: *SEM micrograph (a) and TEM image (b) of EG [micrographs courtesy of XGScience® with permission]*

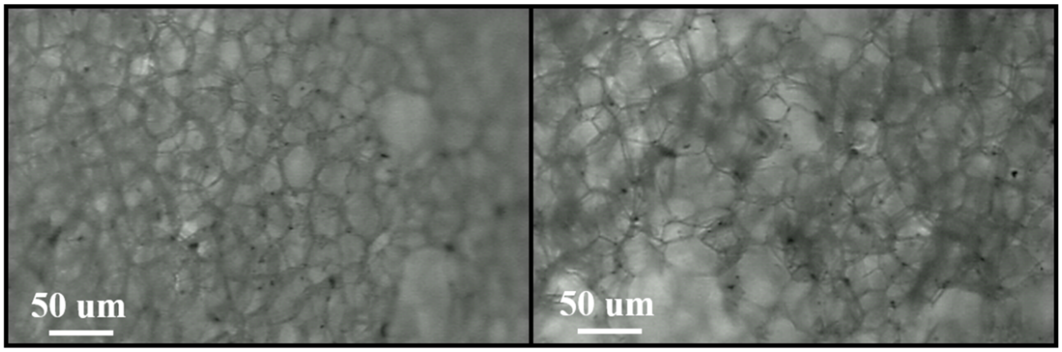

Figure S2: *Optical micrograph of PS/EG foam (black dots are the EG particles).*

Bulk Solid Conductivity

Table S1: *Bulk solid thermal conductivity ($mW \cdot m^{-1} \cdot K^{-1}$) of the composites*

Filler content (wt%)	PS/EG	PS/CNT
0	180	180
0.1	183	185
0.25	189	193
0.5	199	206
1.0	210	222
2.0	250	224

Table S2: *Fitting parameters of the linear regression presented in Figure 5*

Filler content (wt%)	EG		CNT	
	Slope a	Intercept b	Slope a	Intercept b
**0.0**	0.3421	2.394	0.3421	2.394
**0.1**	0.0880	2.010	0.1828	2.000
**0.25**	0.0373	1.520	0.1366	1.500
**0.5**	0.0301	0.901	0.0884	1.200
**1.0**	0.0143	0.901	0.0492	0.859
**2.0**	0.0078	0.892	0.0216	0.900

Effect of Reduced Gas Conduction on the Total Thermal Conductivity in Composite Foams

In this study, the contribution from heat conduction through the gas to the total thermal conductivity PS/EG foams is remarkably high in the range of 65% to 80% because of a large volume fraction of the gas at high expansion. The total thermal conductivity of PS/EG foams could be further decreased by using a low thermal conductive gas such as S-propene with a low thermal conductivity of 10.2 $mW{\cdot}m^{-1}{\cdot}K^{-1}$. The advantages of using S-propene are two-fold: (1) its low bulk gas conductivity and (2) formation of a large foam expansion level caused by its strong plasticization effect on PS. In addition, the contribution from heat conduction through the solid in largely expanded foams is limited due to a low solid fraction (i.e., high gas fraction). Thus, incorporation of the S-propene gas in producing largely expanded foams reduced both the solid conductivity and gas conductivity of PS/EG foams.

Because of the unknown S-propene contents remaining in the cells after foaming [S1], the gas conductivity of the S-propene foams could not be accurately calculated. It was then *estimated* by subtraction of the experimentally measured total thermal conductivity from the known solid and radiative conductivities.

With the use of S-propene in producing PS/EG-A foams, ~35-fold (neat PS) and ~50-fold (PS/EG-A 2.0 wt%) volume expansion ratios have been obtained. Because of the high IR absorption property of EG-A, as Figure S3 shows, increasing the content of EG-A steady reduced the contribution from thermal radiation from 5.1 $mW{\cdot}m^{-1}{\cdot}K^{-1}$ (neat PS) to 1.7 $mW{\cdot}m^{-1}{\cdot}K^{-1}$ (PS/EG-A 2.0 wt%). At high expansion, the low solid conductivity of 2.2–3.1 $mW{\cdot}m^{-1}{\cdot}K^{-1}$ is achieved. The total thermal conductivity of PS/EG and S-propene co-blowing agent, was reduced to a minimum of 21.5 $mW{\cdot}m^{-1}{\cdot}K^{-1}$, much lower than other composite foams. It is worth noting that since the S-propene gas slowly diffuses out of the foam, the effect of the S-propene gas is temporary. However, a certain amount of S-propene remains in the closed cells, decreasing the foam thermal conduction. Further study will be conducted to investigate the change of the total thermal conductivity with time.

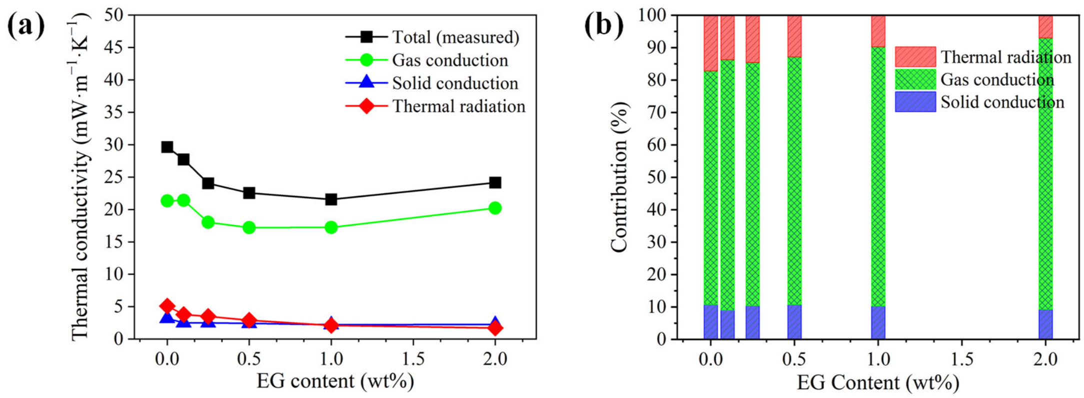

Figure S3: *Analyzed thermal conductivity data of the PS/EG-A samples foamed in $scCO_2$–S-propene at 5.9 MPa. (a) Experimental total thermal conductivities, estimated gas conductivities, calculated solid conductivities, and experimental radiative thermal conductivities; (b) Contribution of each heat-transfer term in percentages to the total thermal conductivity.*

Figure S4 compares the total thermal conductivities of the PS/CNT [S2] , PS/EG, PS/EG-A and PS/EG-B composite foams. At the same filler content (CNT and EG) and volume expansion, the PS/EG composite foams had a lower total thermal conductivity compared to the PS/CNT composite foams, due to the better IR-radiation absorbing attribute of EG than that of CNT. The reduction of gas conduction is highlighted by using the co-blowing agent of S-propene to produce high expansion PS/EG (PS/EG-A and PS/EG-B) foams with ~ 35 to 50-fold volume expansion ratios. As a result, the lowest total thermal conductivity obtained was 19.6 $mW·m^{-1}·K^{-1}$ for PS/EG-B. This outstanding result was attributed to three factors: (1) an effective blocking of IR radiation with the addition of EG (reduced the radiative thermal conductivity in foams with high volume expansion); (2) reduced solid conductivity at high expansion of ~50-fold volume expansion ratio; and (3) the usage of the S-propene co-blowing agent (reduced the gas conductivity).

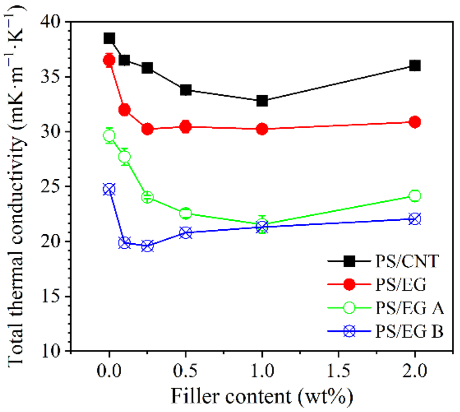

Figure S4: *Comparison of the experimental total thermal conductivities for the PS/CNT foams [S2], the PS/EG foams (foamed in $scCO_2$–pentane at 13.8 MPa), and the PS/EG-A and PS/EG-B foams (foamed in $scCO_2$–S-propene at 5.9 MPa and 6.6 MPa, respectively). Note that all foams have different expansion ratio.*

Heat Transfer Theory though Foams

The total thermal conductivity (λ_{total}) of the polymeric foam with sub-millimeter cells is determined by heat conduction through the gas (λ_{gas}), heat conduction through the solid (λ_{solid}) and the radiative heat transfer (λ_{rad}) as natural convection is negligible [S3] [S4]. Hence, the foam's total thermal conductivity is expressed as follows [S5]:

$$\lambda_{total} = \lambda_{gas} + \lambda_{solid} + \lambda_{rad} \tag{S1}$$

The gas conductivity (λ_{gas}) depends on the gas type and the foam structure in terms of the void fraction ε_{VF} and the cell size d as follows [S6]:

$$\lambda_{gas} = \varepsilon_{VF} \frac{1}{1+2B\frac{\ell_{mean}}{d}} k^0_{gas} \tag{S2}$$

where k^0_{gas} is the bulk gas conductivity (25.7 mW·m^{-1}·K^{-1} for air [S7], 15 mW·m^{-1}·K^{-1} for pentane [S8], and 10.2 mW·m^{-1}·K^{-1} for S-propene [S9]), and B is the energy transfer efficiency between the gas molecules and the cell walls, which is derived from the kinetic theory of gas [S6]:

$$B = \left(\frac{2-\alpha_T}{2\alpha_T}\right)\left(\frac{9\gamma-5}{\gamma+1}\right) \tag{S3}$$

where α_T is the thermal accommodation coefficient ($\alpha_T = 0.9$) and γ is the heat capacity ratio ($\gamma = C_P/C_V = 1.4$). The mean free path of gas molecules l_{mean} can be estimated from the collision theory [S10]:

$$l_{mean} = \frac{k_B T}{\sqrt{2}\pi D_m^2 P} \tag{S4}$$

where k_B is the Boltzmann constant, 1.38×10^{-23} m^2·kg ·s^{-2}·K^{-1}, T is the average absolute temperature, P is the ambient pressure (1.01×10^5 Pa) and D_m is the collision diameter of air (3.8×10^{-10} m). The calculated mean free path of air molecules (l_{mean}) is 64 nm at the ambient temperature and pressure.

Heat conduction through the solid depends on the volume fraction of the solid $(1 - \varepsilon_{VF})$ with ε_{VF} being the void fraction and the foam geometry in term of the strut fraction f_s (i.e. the volume fraction of solid located in struts). For low-density polymeric foams (ε_{VF} > 94%), the solid conductivity can be described by the Glicksman model [S4, S5].

$$\lambda_{solid} = (1 - \varepsilon_{VF})\left(\frac{2-f_s}{3}\right) k^0_{solid} \tag{S5}$$

where k^0_{solid} is the thermal conductivity of bulk solid. For foams with the same thermal conductivity of the solid at a fixed void fraction, heat conduction through the solid is determined by a factor $(2 - f_s)/3$ indicating the influence of the foam geometry. The contribution from heat conduction through the solid decreases when increasing foam expansion (i.e., a higher void fraction) and the strut fraction (i.e., a higher fraction of solid located in struts, indicating more tortuous structure for solid conduction).

The radiative heat transfer in foams can be described by the Rosseland approximation [S11, S12]. The radiative heat transfer is inversely proportional to the overall radiation-blocking property of the foam, as follows.

$$\lambda_{rad} = \frac{16n^2\sigma T^3}{3K_{e,R}} \tag{S6}$$

where n is the effective index of refraction of the foam. For high expansion polymeric foams, n has the same value as air (e.g., 1). σ is Stefan-Boltzmann's constant, 5.67×10^{-8} W·m^{-2}·K^{-4}. At room temperature, over 90% of the radiation energy is contributed from the IR range with 2.5–25 μm wavelengths (based on the Planck's spectral energy distribution), which can be studied using the Fourier Transform Infrared Spectroscopy (FTIR) [S13]. The Rosseland spectral extinction coefficient ($K_{e,R}$) is as follows [S10, S14-S16]:

$$\frac{1}{K_{e,R}} = \frac{\int_0^\infty \frac{1}{K_{e,\lambda}} \frac{\partial e_{b,\lambda}}{\partial T} d\lambda}{\int_0^\infty \frac{\partial e_{b,\lambda}}{\partial T} d\lambda} = \int_0^\infty \frac{1}{K_{e,\lambda}} \frac{\partial e_{b,\lambda}}{\partial e_b} d\lambda \quad \text{(S7)}$$

$$\frac{\partial e_{b,\lambda}}{\partial e_b} = \frac{\pi}{2} \frac{C_1 C_2}{\lambda^6} \frac{\sigma^{1/4}}{e_b^{5/4}} \frac{exp(C_2/\lambda T)}{[exp(C_2/\lambda T) - 1]^2} \quad \text{(S8)}$$

$$e_b = \sigma T^4 \quad \text{(S9)}$$

where the constants C_1 and C_2 are 3.74×10^{-16} W·m^2 and 1.44×10^4 μm·K, respectively. $e_{b,\lambda}$ is the local spectral energy per unit wavelength and e_b is the overall emissive power of blackbody radiation. The spectral extinction coefficient $K_{e,\lambda}$ indicates the spectral radiation-blocking property per unit thickness of the foam sample, and it can be obtained by analyzing the spectral IR transmittance ($\tau_{n,\lambda}$) through the foam, as follows [S17, S18]:

$$\tau_{n,\lambda} = c e^{(-\int_0^L K_{e,\lambda} dx)} \quad \text{(S10)}$$

where L is the foam sample thickness, c is a constant. By the linear regression of $ln(\tau_{n,\lambda})$ against L, the slope of the straight line is $K_{e,\lambda}$.

Strut fraction

In highly expanded foam, the cells, cell walls and struts can be regarded as polyhedrons, thin slabs, and triangular prisms, respectively. The volume occupied by a cell (V_{cell}), the cell struts (V_{struts}) and the cell walls (V_{walls}) are calculated according to references [S2, S19] as follows:

$$V_{cell} = 0.35\Phi_c^3 \quad \text{(S11)}$$

$$V_{struts} = 2.8\Phi_s^2\Phi_c - 3.93\Phi_s^3 \quad \text{(S12)}$$

$$V_{walls} = \frac{\rho_f}{\rho_s} 0.35\Phi_c^3 - 2.8\Phi_s^2\Phi_c + 3.93\Phi_s^3 \quad \text{(S13)}$$

where V_{cell}, V_{struts}, and V_{walls} are the cell volume, the strut volume, and the cell wall volume, respectively. Φ_c and Φ_s are the cell size and the strut diameter, respectively. For each sample, three SEM micrographs at three different positions were analyzed to identify Φ_c and Φ_s. Then, the strut fraction (f_s) was calculated, as follows [S19]:

$$f_s = \frac{V_{struts}}{V_{struts} + V_{walls}} \quad \text{(S14)}$$

References

[S1] Park CB, Behravesh AH, Venter RD. Low density microcellular foam processing in extrusion using CO_2. Polym Eng Sci. 1998;38(11):1812-23.

[S2] Gong P, Buahom P, Tran M-P, Saniei M, Park CB, Pötschke P. Heat transfer in microcellular polystyrene/multi-walled carbon nanotube nanocomposite foams. Carbon. 2015;93:819-29.

[S3] Glicksman LR. Heat transfer in foams. Low Density Cellular Plastics: Physical Basis of Behavior, 1st Edition: Chapman & Hall, London, UK; p. 104-52.
[S4] Glicksman LR, Torpey M. Factors governing heat transfer through closed cell foam insulation. J Build Phys. 1989;12(4):257-69.
[S5] Schuetz MA, Glicksman LR. A basic study of heat transfer through foam insulation. J Cell Plast. 1984;20(2):114-21.
[S6] Ferkl P, Pokorný R, Bobák M, Kosek J. Heat transfer in one-dimensional micro- and nano-cellular foams. Chem Eng Sci. 2013;97:50-8.
[S7] B. Vargaftik N. Handbook of thermal conductivity of liquids and gases: Taylor & Francis; 1993.
[S8] Limited RT. Blowing agent systems: Formulations and processing: Rapra technology Ltd, Shawbury, Shrewsbury, UK, 19th February 1998: Rapra Technology; 1994.
[S9] Honeywell. Solstice 1233zd (E) trans-1-chloro-3,3,3-trifluoropropene. Technical Information 2013(Performance Materials and Technologies 101 Columbia Road, Morristown, NJ 07960 USA).
[S10] Zhao CY, Lu TJ, Hodson HP. Thermal radiation in ultralight metal foams with open cells. Int J Heat Mass Transfer. 2004;47(14):2927-39.
[S11] Alvarez-Lainez M, Rodríguez-Pérez MA, De Saja JA. Thermal conductivity of open-cell polyolefin foams. J Polym Sci, Part B: Polym Phys. 2008;46(2):212-21.
[S12] Campo-Arnáiz RA, Rodríguez-Pérez MA, Calvo B, de Saja JA. Extinction coefficient of polyolefin foams. J Polym Sci, Part B: Polym Phys. 2005;43(13):1608-17.
[S13] Wei G, Liu Y, Zhang X, Du X. Radiative heat transfer study on silica aerogel and its composite insulation materials. J Non-cryst Solids. 2013;362:231-6.
[S14] Baillis D, Raynaud M, Sacadura JF. Spectral radiative properties of open-cell foam insulation. J Thermophys Heat Transfer. 1999;13(3):292-8.
[S15] Glicksman L, Schuetz M, Sinofsky M. Radiation heat transfer in foam insulation. Int J Heat Mass Transfer. 1987;30(1):187-97.
[S16] Kuhn J, Ebert HP, Arduini-Schuster MC, Büttner D, Fricke J. Thermal transport in polystyrene and polyurethane foam insulations. Int J Heat Mass Transfer. 1992;35(7):1795-801.
[S17] Wu J, Sung W, Chu H. Thermal conductivity of polyurethane foams. Int J Heat Mass Transfer. 1999;42(12):2211-7.
[S18] Tseng C, Kuo K. Thermal radiative properties of phenolic foam insulation. J Quant Spectrosc Radiat Transfer. 2002;72(4):349-59.
[S19] Placido E, Arduini-Schuster MC, Kuhn J. Thermal properties predictive model for insulating foams. Infrared Phys Techn. 2005;46(3):219-31.
